# The Impact of Commonly Used Medications on Erectile Dysfunction: Which Drugs Deserve Particular Attention?

**DOI:** 10.7759/cureus.93259

**Published:** 2025-09-26

**Authors:** Bartosz Czyzewski, Joanna Czyzewska, Alicja Dorota, Michal Dorota, Karol Kozlowski, Wojciech Zywiec, Cezary Milczarek, Anna Mariankowska, Illia Koval

**Affiliations:** 1 Department of Internal Medicine, Central Clinical Hospital of the Medical University of Lodz, Łódź, POL; 2 Student Scientific Club of Biomedicine and Experimental Surgery, Faculty of Medicine, Medical University of Lodz, Łódź, POL; 3 Department of Internal Medicine, Zaglebiowskie Oncology Center in Dąbrowa Górnicza, Dąbrowa Górnicza, POL; 4 Conservative Dentistry, Central Clinical Hospital of Medical University of Lodz, Łódź, POL; 5 Anesthesia and Critical Care, Provincial Integrated Hospital, Kielce, POL; 6 General Medicine, Provincial Hospital of St. Luke, Tarnów, POL; 7 Medicine, Provincial Hospital in Poznań, Poznań, POL

**Keywords:** antihypertensive drugs, antipsychotics, beta-blockers, erectile dysfunction, medication side effects, sexual dysfunction

## Abstract

Erectile dysfunction (ED) is a common but often underrecognized side effect of numerous medications prescribed for chronic conditions, particularly cardiovascular, psychiatric, and neurological disorders. This article reviews the impact of various drug classes on erectile function, focusing on mechanisms and clinical implications. Antipsychotics, widely used in schizophrenia, frequently cause ED through dopamine inhibition and increased prolactin levels, affecting libido and erectile capacity. Beta-blockers, especially older non-selective agents, contribute to ED by causing vascular constriction and hormonal alterations, while newer agents like nebivolol may improve erectile function via nitric oxide release. Diuretics, particularly thiazides, show mixed evidence regarding their effect on erectile function, though aldosterone receptor antagonists such as spironolactone are linked to antiandrogenic side effects. Centrally acting antihypertensives, antiepileptics, non-steroidal anti-inflammatory drugs (NSAIDs), lithium, and opioids also play significant roles in sexual dysfunction, primarily through hormonal disruption, vascular effects, and central nervous system pathways. Notably, opioids exert profound effects on the hypothalamic-pituitary-gonadal axis, leading to high rates of ED in younger men. In contrast, renin-angiotensin system inhibitors and calcium channel blockers generally exhibit neutral or beneficial effects on sexual function. Understanding these drug-related risks is crucial for clinicians to tailor therapies that minimize sexual side effects, thereby enhancing patient adherence and overall quality of life. Further research is warranted to clarify mechanisms and develop effective management strategies for medication-induced ED.

## Introduction and background

Erectile dysfunction (ED), defined as the persistent inability to reach or maintain a penile rigidity enough for sexual satisfaction, is the most prevalent male sexual disorder and a significant public health concern [1-2]. It affects men across all age groups, with its prevalence increasing with age and the presence of comorbid conditions such as hypertension, coronary artery disease, heart failure, diabetes mellitus, hormonal dysfunction, obesity, dyslipidemia, and depression [3-8]. Epidemiological data suggest that ED affects approximately 10-52% of men worldwide, with over 30 million men in the United States and nearly 100 million globally experiencing some degree of this condition [9-10].

The physiological mechanism underlying penile erection involves a complex interplay of neural, vascular, hormonal, and psychological factors. A key component in this process is endothelial-derived nitric oxide (NO), which promotes vasodilation, increases blood flow to the penile corpora cavernosa, and enables the veno-occlusive mechanism necessary to sustain an erection. Disruption of this pathway, often due to endothelial dysfunction and oxidative stress, leads to impaired erectile function [5,11-14].

ED not only compromises physical health but also exerts a substantial psychological burden on affected individuals, negatively impacting self-esteem, interpersonal relationships, and overall quality of life. Sexual health is an integral aspect of general well-being, and sexual dysfunction can significantly reduce life satisfaction [15-17].

While ED has multifactorial origins, including vascular, neurological, endocrine, psychological, and anatomical causes, pharmacological factors are increasingly recognized as significant contributors. Numerous medications commonly prescribed for chronic conditions, such as antihypertensives, antidepressants, and antipsychotics, have been associated with ED [5,18-19]. In some cases, drug-induced ED may lead to poor adherence or discontinuation of essential treatments, especially in patients with hypertension or mental health disorders [20].

Given the aging population and rising prevalence of chronic illnesses, the potential iatrogenic impact of medications on erectile function warrants closer examination. Importantly, identifying and modifying culprit drugs, when clinically feasible, can alleviate symptoms and improve both sexual function and adherence to treatment regimens [21-22].

This review summarizes current evidence on the impact of commonly used medications on erectile function. It highlights which drugs most frequently contribute to ED to guide treatment modifications that may reduce adverse sexual effects. The ultimate goal is to support clinicians in making informed decisions when managing comorbidities in patients with ED, optimizing treatment while minimizing adverse sexual effects.

The literature for this review was retrieved from PubMed, Scopus, Google Scholar, and other databases, with the search covering studies published through May 2025.

## Review

Medications contributing to erectile dysfunction

Medication-related side effects are estimated to account for up to 25% of all ED cases. Among the most commonly implicated drug classes are antihypertensives, antidepressants, and antipsychotics, particularly in older patients with multiple comorbidities. Although the exact mechanisms vary and are often poorly understood, these medications may interfere with vascular, neural, or hormonal pathways critical to normal erectile function [5, 18-19].

Importantly, the potential for drug-induced ED should always be assessed prior to initiating extensive diagnostic evaluation or therapy. In patients with active sexual lives, clinicians should consider selecting medications with a lower risk of sexual side effects. Adjusting or replacing the offending agent, when clinically appropriate, can significantly improve erectile function and overall quality of life [20, 22].

While chronic diseases such as diabetes and hypertension are known contributors to ED, it is increasingly recognized that ED itself may be an early marker of underlying cardiovascular or metabolic disease [19]. Therefore, identifying and addressing medication-related ED is not only essential for sexual health but may also aid in the broader management of systemic health risks [21].

Antidepressants

Antidepressants, particularly selective serotonin reuptake inhibitors (SSRIs), are among the most frequently reported medications associated with sexual dysfunction. While SSRIs are primarily known to affect libido and orgasm, they can also contribute to ED [23].

SSRIs increase serotonin (5-HT) levels in the synaptic cleft, which may inhibit dopamine pathways critical for sexual arousal and erection. Elevated serotonin levels can also lead to increased prolactin secretion through dopaminergic suppression, further exacerbating sexual side effects. Furthermore, serotonin has been shown to induce penile smooth muscle contraction via 5-HT1A, 5-HT2A, and 5-HT4 receptors, potentially impairing cavernosal blood flow and erection [23-24].

Although some studies, such as randomized trials with citalopram and fluoxetine, found no objective decline in erectile function, subjective complaints were common [25].

The incidence of SSRI-induced sexual dysfunction is high, estimated between 50-70%, and can affect all stages of the sexual response cycle, including desire, arousal, and orgasm [26-30]. Notably, drugs that predominantly affect dopamine or norepinephrine pathways, or that block specific serotonin receptors (e.g., 5-HT2), are associated with a lower risk of sexual side effects [31].

Tricyclic antidepressants (TCAs) and monoamine oxidase inhibitors (MAOIs) are also linked to ED, with multivariable analyses showing increased risk (e.g., TCAs: OR = 3.35). Surveys like Boston Area Community Health (BACH) and National Health and Nutrition Examination Survey (NHANES) report a higher risk of ED among users of TCAs and benzodiazepines [5, 32-33].

In contrast, certain newer antidepressants, such as bupropion and vortioxetine, which primarily enhance dopamine and norepinephrine activity or modulate serotonergic pathways, appear to have a lower risk of inducing ED and may even improve sexual function in some cases [31, 34].

Given the widespread use of antidepressants in the treatment of mood and anxiety disorders, awareness of their potential sexual side effects is essential. When treating patients with coexisting ED and depression, clinicians should carefully consider the choice of antidepressant, balancing psychiatric efficacy with the risk of sexual dysfunction [35].

Benzodiazepines

Benzodiazepines (BZDs) have been associated with various forms of sexual dysfunction (SD), including decreased libido and ED. The prevalence and severity of these effects appear to be dose- and duration-dependent, with long-term use posing a higher risk. Their effect is primarily mediated through enhanced GABA-A receptor activity, which suppresses central arousal and reduces penile erection [36]. Additional mechanisms may include alterations of the hypothalamic-pituitary-gonadal axis, leading to reduced testosterone, as well as sedation and reduced psychogenic stimulation. Moreover, the combination of these pharmacologic effects with underlying psychiatric conditions, such as anxiety or depression, may amplify the risk of SD.

Multivariable analyses have shown that BZDs are significantly associated with ED (adjusted OR = 2.34). Data from the BACH survey indicate that among commonly used medications, psychoactive agents, especially BZDs and tricyclic antidepressants, are among the most strongly linked to increased ED risk, with a two- to threefold increase in odds [32]. This highlights the importance of monitoring sexual side effects and considering alternative therapies when feasible.

Antipsychotics

SD, including ED, is a prevalent and clinically significant adverse effect of antipsychotic treatment, particularly among individuals with schizophrenia [31,37-38]. Reports suggest that between 48% and 75% of patients on antipsychotic medications experience some form of SD, which can lead to reduced quality of life and poor treatment adherence [39-44].

The primary mechanism by which antipsychotics induce ED is through the inhibition of dopaminergic pathways and the subsequent increase in prolactin levels. Dopamine plays a critical role in modulating sexual arousal and function; its inhibition, common across both typical and atypical antipsychotics, can severely disrupt erectile capacity. Additionally, many antipsychotics act on other receptors involved in sexual function, including serotonergic (5-HT2), adrenergic (α1), histaminergic (H1), and muscarinic (M1) receptors, contributing further to dysfunction [23, 45-47].

While the medications themselves are a significant factor, it's worth noting that psychotic disorders may also independently affect sexual function, complicating the clinical picture [48].

Among antipsychotics, aripiprazole appears to have a more favorable sexual side effect profile. Studies have shown that switching to or adding aripiprazole to an existing regimen can reduce the incidence of ED and improve overall sexual function. Its partial agonist activity at dopamine D2 receptors may underlie this benefit [49-50].

Overall, while antipsychotic therapy is essential in managing schizophrenia and related disorders, its impact on sexual health, affecting erection, desire, ejaculation, orgasm, and satisfaction, should not be overlooked. Careful selection and individualized treatment adjustments, such as switching to aripiprazole, may help mitigate these adverse effects [38,50].

Antiepileptic Drugs

Antiepileptic drugs (AEDs) are commonly associated with ED, often through hormonal and neuroendocrine mechanisms. Enzyme-inducing AEDs can increase sex hormone-binding globulin (SHBG), reducing free testosterone levels. They also accelerate the metabolism of sex hormones and may suppress the hypothalamic-pituitary-gonadal axis, leading to hypogonadotropic hypogonadism. Additionally, AEDs may affect serotonergic pathways involved in sexual function [51-54]. These combined hormonal and neurochemical effects can disrupt multiple stages of the sexual response cycle, including desire, arousal, and orgasm. Studies have shown increased risk of ED in patients on valproate or levetiracetam [55]. While hormonal disruption is a likely contributor, the role of epilepsy itself and psychosocial factors remains to be fully clarified [56]. Factors such as seizure frequency, comorbid depression, and social stressors may further exacerbate SD, highlighting the need for a comprehensive approach when assessing ED in these patients.

Beta-Blockers

ED is commonly observed in hypertensive patients, particularly those treated with beta-blockers (BBs). Although BBs are effective in managing hypertension, coronary artery disease, and heart failure, they are frequently associated with impaired sexual function [13, 57-58].

BBs can impair erection by reducing penile blood flow through vasoconstriction and lowering testosterone levels. The incidence of ED varies depending on the type of beta-blocker. Older BBs, such as the non-selective propranolol and the β1-selective atenolol, are more likely to cause ED, partly due to vasoconstriction and reduced testosterone levels. In contrast, newer BBs with vasodilating properties, like carvedilol and especially nebivolol, have a lesser impact or may even improve erectile function. Nebivolol, a third-generation beta-1 selective blocker, enhances NO release, promoting penile vasodilation [10, 59-62].

In a study by Cordero et al., the rates of ED among beta-blocker users ranged from 28.8% with atenolol to just 3.4% with metoprolol [63]. While metoprolol appears to have a minimal effect, nebivolol stands out for its potential benefits on erectile function [60, 62].

Despite some conflicting findings, BBs, especially the older types, remain among the antihypertensive agents most commonly linked to ED [64].

Diuretics

Thiazide diuretics have historically been linked to ED, likely due to sodium depletion stimulating central α-adrenergic pathways. Early studies suggested up to a 2.4-fold increased risk of ED [65-67]. However, larger studies like TOMHS and recent meta-analyses failed to show a consistent association. While thiazides may affect erectile function in some individuals, current evidence does not strongly support a significant risk [64, 68-70].

Spironolactone, a potent aldosterone receptor antagonist with antiandrogenic properties, has been associated with ED, reduced libido, and gynecomastia [71]. These effects are likely due to interference with androgen activity and possible suppression of gonadotropin secretion [67]. ED has been reported in up to 5.8% of men using spironolactone [72-73]. In contrast, eplerenone, a newer, more selective mineralocorticoid receptor antagonist, appears to have a lower risk of sexual side effects [71]. Its higher receptor selectivity reduces antiandrogenic activity, making it a preferable option in patients at risk of ED.

Central-Acting Antihypertensive Drugs

Centrally acting antihypertensives such as clonidine and α-methyldopa are associated with a high prevalence of ED.

These agents exert their action at the level of the central nervous system, where modulation of adrenergic tone directly interferes with neural circuits responsible for sexual arousal. Their mechanism involves central inhibition of sympathetic outflow by stimulating α-adrenergic receptors or depleting catecholamines, which impairs vasoconstrictor and cardioacceleratory pathways. Such alterations in autonomic balance not only reduce penile rigidity but may also blunt libido and orgasmic response, leading to a broader spectrum of SD.

ED has been reported in up to 70% of patients treated with clonidine and 53% with methyldopa [64, 74-76]. The high prevalence underscores the importance of evaluating sexual side effects when these agents are prescribed, particularly in men with preexisting sexual concerns.

Non-Steroidal Anti-Inflammatory Drugs (NSAIDs)

Regular non-steroidal anti-inflammatory drug (NSAID) use, including aspirin, has been increasingly linked to ED. The risk appears to be dose- and duration-dependent, with chronic use conferring the greatest likelihood of SD. Multiple cohort and community studies, such as the PCPT and BACH surveys, report a significantly higher incidence of ED in men regularly taking NSAIDs [32,77-80]. Aspirin, in particular, has shown a strong association, potentially due to its inhibition of the COX pathway, which reduces vasodilatory prostaglandins and disrupts NO synthesis, both crucial for erection [81-82]. This impairment of endothelial function may lead to decreased penile blood flow and compromised veno-occlusive mechanisms, contributing to persistent erectile difficulties. The observed odds ratios suggest up to a 2.4-fold increased risk, indicating NSAIDs as a notable, though often overlooked, contributor to ED [77-78]. Given their widespread use for chronic pain and cardiovascular prophylaxis, awareness of this potential adverse effect is clinically important for patient counseling and management.

Lithium

Lithium, commonly prescribed for bipolar disorder, has been associated with SD, including ED, in multiple clinical reports. Patients receiving lithium, whether as monotherapy or in combination with other psychotropic drugs, frequently report ED [83]. Mechanisms are not fully elucidated but may involve lithium’s effects on neuroendocrine signaling, neurotransmitter modulation, and hormonal balance, which can interfere with normal erectile physiology. Interestingly, a randomized placebo-controlled study found that adjunctive aspirin therapy significantly improved lithium-induced ED, highlighting a potential role for anti-inflammatory modulation in mitigating these side effects and pointing toward new avenues for therapeutic intervention [82].

Digoxin

Digoxin, frequently prescribed for heart failure, has been associated with ED in several observational studies. Although data are limited, the relationship appears clinically significant, particularly in men receiving long-term therapy. The exact mechanism remains unclear, but potential explanations include hormonal alterations, such as reduced testosterone and luteinizing hormone levels, and increased estrogen, which together may disrupt the normal hormonal balance required for sexual function. Additionally, impaired NO-mediated smooth muscle relaxation due to sodium pump inhibition in the corpora cavernosa can compromise penile blood flow and the veno-occlusive mechanism, further contributing to erectile difficulties [84-86].

Opioids

Chronic opioid use is strongly associated with ED, with prevalence rates reaching 21-52% among users, often at younger ages than in the general population [87-90]. This high prevalence underscores the significant impact of opioids on male sexual health, particularly in long-term therapy. Opioids disrupt the hypothalamic-pituitary-gonadal (HPG) axis, leading to reduced testosterone levels and impaired NO synthesis, both crucial for normal erectile function. The resulting hormonal imbalance is compounded by potential alterations in central and peripheral nervous system signaling. This hormonal imbalance, combined with possible effects on central and peripheral nervous systems, results in diminished libido, arousal, erectile ability, and overall sexual satisfaction [91-94]. While all opioids pose this risk, short-acting and partial agonists may have a milder impact. In addition, gradual dose adjustment and opioid-sparing strategies can sometimes mitigate SD, highlighting the importance of individualized management. Notably, men in treatment with buprenorphine have shown improvements in sexual function over time [90].

Other Drugs

Angiotensin-converting enzyme inhibitors (ACEIs), angiotensin receptor blockers (ARBs), and calcium channel blockers (CCBs) generally show a neutral or even beneficial impact on erectile function. Meta-analyses and trials, including the TOMHS study and recent network reviews, found no significant increase in ED rates with these medications [64, 69-70]. While CCBs appear to have a neutral effect, some data suggest ARBs may improve overall sexual activity, though not necessarily erectile function itself [95].

## Conclusions

ED is a common side effect of many medications used to treat physical and mental health conditions. Drugs like β-blockers, diuretics, antipsychotics, opioids, and NSAIDs can impair erectile function through hormonal, vascular, and neurological mechanisms. Some newer medications, such as nebivolol and certain renin-angiotensin system inhibitors, have fewer negative effects. Recognizing and managing medication-induced ED is important to improve patients’ quality of life and treatment adherence.
